# Temperature-dependent Raman investigation of rolled up InGaAs/GaAs microtubes

**DOI:** 10.1186/1556-276X-7-594

**Published:** 2012-10-26

**Authors:** Raul D Rodriguez, Evgeniya Sheremet, Dominic J Thurmer, Daniel Lehmann, Ovidiu D Gordan, Falko Seidel, Alexander Milekhin, Oliver G Schmidt, Michael Hietschold, Dietrich RT Zahn

**Affiliations:** 1 Semiconductor Physics, Chemnitz University of Technology, Chemnitz, D-09107, Germany; 2 Material Systems for Nanoelectronics, Chemnitz University of Technology, Chemnitz, D-09107, Germany; 3 Institute for Integrative Nanosciences, IFW Dresden, Helmholtzstrasse 20, Dresden, D-01069, Germany; 4 Institute of Semiconductor Physics, Lavrentjev Av. 13, Novosibirsk, 630090, Russia; 5 Solid Surfaces Analysis Group, Chemnitz University of Technology, Chemnitz, D-09107, Germany

**Keywords:** Rolled up tubes, Microtubes, Raman spectroscopy defects, Raman imaging, Strain imaging, Gallium arsenide, Dependent Raman spectroscopy, Gallium arsenide TO phonon

## Abstract

Large arrays of multifunctional rolled-up semiconductors can be mass-produced with precisely controlled size and composition, making them of great technological interest for micro- and nano-scale device fabrication. The microtube behavior at different temperatures is a key factor towards further engineering their functionality, as well as for characterizing strain, defects, and temperature-dependent properties of the structures. For this purpose, we probe optical phonons of GaAs/InGaAs rolled-up microtubes using Raman spectroscopy on defect-rich (faulty) and defect-free microtubes. The microtubes are fabricated by selectively etching an AlAs sacrificial layer in order to release the strained InGaAs/GaAs bilayer, all grown by molecular beam epitaxy. Pristine microtubes show homogeneity of the GaAs and InGaAs peak positions and intensities along the tube, which indicates a defect-free rolling up process, while for a cone-like microtube, a downward shift of the GaAs LO phonon peak along the cone is observed. Formation of other type of defects, including partially unfolded microtubes, can also be related to a high Raman intensity of the TO phonon in GaAs. We argue that the appearance of the TO phonon mode is a consequence of further relaxation of the selection rules due to the defects on the tubes, which makes this phonon useful for failure detection/prediction in such rolled up systems. In order to systematically characterize the temperature stability of the rolled up microtubes, Raman spectra were acquired as a function of sample temperature up to 300°C. The reversibility of the changes in the Raman spectra of the tubes within this temperature range is demonstrated.

## Background

Self-positioning nanostructures with controlled composition and size compatible with mass-production fabrication techniques have been studied for almost two decades, giving rise to the so-called smart tubes in exciting forms such as nanojets [1,2]. The versatility in controlling the size and composition of the smart tubes has made them attractive candidates for applications ranging from spintronics [3] to designing novel substrates for cell adhesion [4]. However, apart from the work of Deneke et al. [5], not much attention has been devoted to the questions concerning how GaAs/InGaAs smart tubes collapse and the role of the TO phonon on identifying defective tubes. In the present work, we also investigate the temperature stability of these rolled up structures by measuring the LO phonon modes of GaAs and InGaAs *in situ* while heating the structures up to 300°C.

## Methods

**Figure 1:**
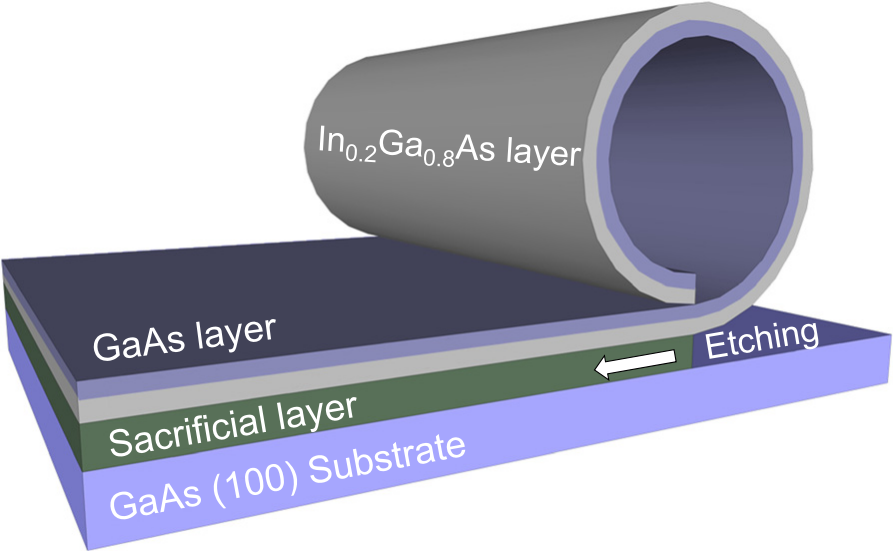
**Sketch of the GaAs/InGaAs smart tubes.** The selective etching of the sacrificial layer promotes the self-rolling process of the two top most layers due to the lattice mismatch between them.

The samples were grown by molecular beam epitaxy. First, the GaAs (100) substrate was deoxidized at roughly 620°C under UHV. Thereafter, a GaAs buffer layer of 150 nm thickness was grown under RHEED observation in order to improve the crystal quality. Next, a 20 nm thick AlAs sacrificial layer was deposited followed by a 10 nm film of In_0.2_Ga_0.8_As and 30 nm film of GaAs. A sketch of the rolled up tubes is shown in Figure 1. The selective etching of the AlAs sacrificial layer provides the driving force for rolling up the tube due to the lattice mismatch between the InGaAs and the GaAs layers. Raman measurements were performed in the backscattering geometry using the 514.5 nm line of an Ar^+^ laser and the 632.8 nm line of a HeNe laser. The Raman spectrometer is a LabRam HR800 (HORIBA Jobin Yvon GmbH, Bensheim, Germany) with an optical microscope Olympus BX40 (Olympus Corporation, Hamburg, Germany). A 100× objective (N.A. 0.9) was used to illuminate the sample and to collect the Raman signal, giving a diffraction limited resolution of 0.61∙*λ*/N.A ≈ 349 nm (*λ* = 514.5 nm). The size of the laser spot on the sample has been experimentally determined using the scanning knife-edge method giving a value of 488 ± 50 nm for a confocal hole aperture of 100 μm (*λ* = 514.5 nm). A liquid nitrogen-cooled back-illuminated charge-coupled device was used for the detection of the Raman signal using a diffraction grating of 600 l/mm and a spectral resolution of 2 cm^−1^. The laser power was limited to the range from 0.05 to 0.3 mW for green and red lasers, respectively, in order to prevent damage of the tubes. Above these laser intensities, we observed oxidation of the tubes as evidenced by their Raman signal. Full Raman spectra were acquired by Raman imaging with a step size of 500 nm. Temperature controlled experiments were performed by placing the sample on a Linkam TC91 stage (Linkam Scientific Instruments Ltd., Surrey, England). The temperature was changed from room temperature up to 300°C.

## Results and discussion

### Strain made visible by Raman spectroscopy imaging

**Figure 2:**
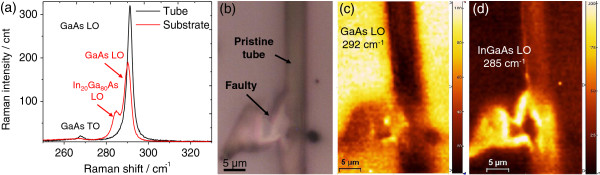
**Raman spectra and images.** (**a**) Raman spectra on the InGaAs/GaAs system with 514.5 nm laser line. TO and LO phonons appear on the substrate, while a red shift of the GaAs LO mode occurs in the tube. The InGaAs LO from the tube bilayer is observed at 285 cm^−1^. (**b**) The microscopic image of a faulty tube shown in (**c**) and (**d**). Two Raman images are displayed: (**c**) the intensity of unstrained LO phonon mode of the GaAs substrate and (**d**) the Raman intensity of the LO mode of InGaAs showing a pristine tube and misfolded tube regions.

The Raman spectra of GaAs/InGaAs tubes show characteristic first order phonon modes in the range from 250 to 300 cm^−1^ as shown in Figure 2a. The strongest features visible in the Raman spectra are the LO mode of the GaAs and the LO mode of InGaAs in the tube bilayer. The LO phonon is red-shifted in the tube due to residual strain in the tube with respect to the substrate. The shift of this mode can be used to quantify the residual stress and the diameter of the tube, which is also determined by the amount of initial strain [6,7]. The TO mode of GaAs at 268 cm^−1^, although of low intensity compared to the LO mode, is still visible on the substrate area. However, the TO mode is hardly visible on the tube. The selection rules forbid TO phonons for this geometry, although the high N.A. objective used has wide collection angle and leads to deviation from perfect backscattering geometry and can thus induce the appearance of the TO mode.

Acquiring Raman spectra across a sample in a point-wise manner allows spatial sample heterogeneities arising from variations in physico-chemical properties. These can be made visible by selecting relevant Raman shifts and plotting their intensities as color-coded pictures (Figures 2b,c,d). Individual mapping of the unstrained LO GaAs (InGaAs) mode intensity allows a clear visualization of the substrate (the faulty region of the tube shown in Figure 2b). In this section, we aim at identifying common signatures in faulty rolled up tubes. These tubes are those lacking the uniform cylindrical symmetry of perfect roll-ups, and their analysis might give clues about the origin of the structural failure of the tubes as well as their early detection using Raman spectroscopy.

### TO phonons: the signature of faulty structures

**Figure 3:**
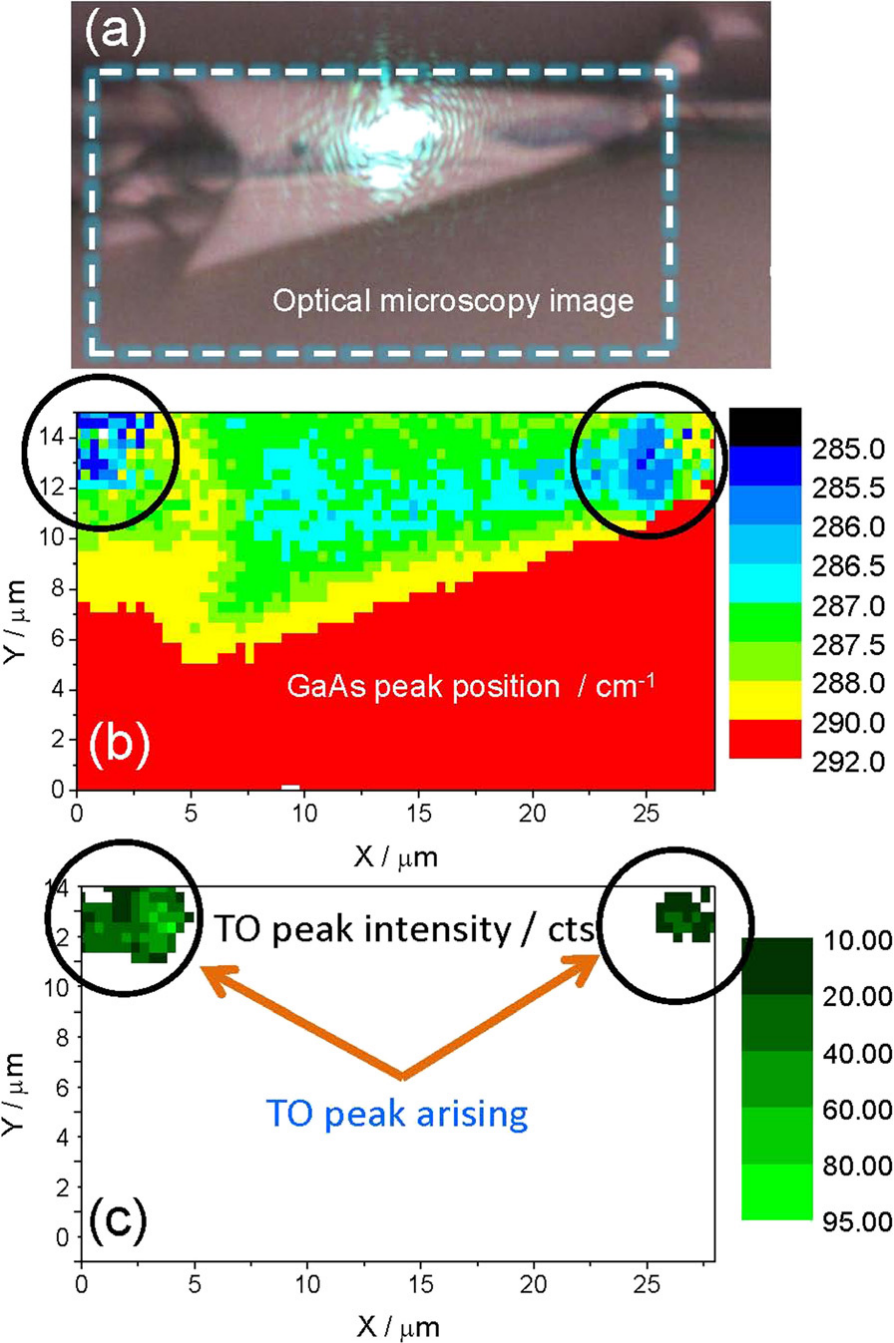
**Raman mapping.** The GaAs peak position around a defective microtube (cone) acquired with 514.5 nm laser line. (**a**) Optical micrograph surrounded by the dashed line where the Raman images (**b**) and (**c**) shown in the figure were taken. In (**b**), the Raman image shows the peak position of the GaAs LO phonon mode. (**c**) Raman image of the TO mode intensity of GaAs.

Careful examination of a second faulty structure (a cone) shown in Figure 3a indicates highly defective GaAs regions at the two ends of the cone and a strongly shifted GaAs LO peak in Figure 3b.

**Figure 4:**
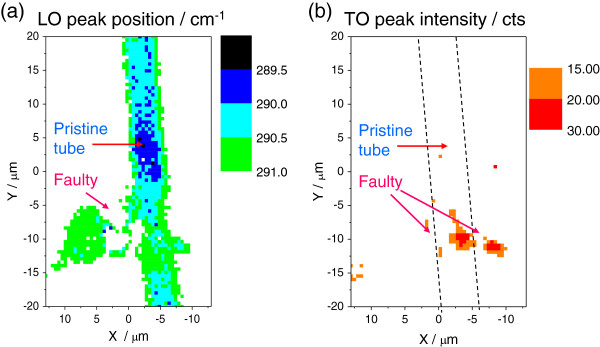
**Analysis of the Raman imaging of the faulty structure shown in Figure **2**.** (**a**) Peak position of the GaAs LO mode of the tube. The TO mode Raman intensity map is shown in (**b**).

We attribute a low LO peak position in Figure 3b to strong heating of the cone due to illumination with the laser light. While, if a shift would originate from inhomogeneous strain, then such strain in the layer should exceed 8%, which is hard to believe for the case of a crystalline material. Using the calibration of the peak position vs. temperature as discussed in the following section, the temperature in the cone-like structure can be estimated as 150°C higher than on the pristine tubes measured on different tubes on the same sample. No change in color, which would be an indication of sample burning, was observed after illumination. Moreover, we observed only a very weak Raman signal of TO peak on pristine tubes as shown in Figure 2a, though relaxation of selection rules could be expected for rolled up structures since they do not have a (100) flat surface. However, we have found intensity of the TO phonon mode comparable to the intensity of the LO mode in the regions with lowest Raman shift at the edges of the cone (Figure 3c). We speculate that the appearance of the TO mode is directly related with the faulty regions of the tube due to a further relaxation of the selection rules as a consequence of the decrease in crystal symmetry (defects) [8]. In order to test this hypothesis, we return to the Raman data of the sample shown in Figure 2b, but not with an emphasis on the peak intensity but on the peak position of the LO mode in GaAs and special emphasis on the TO mode. The results of the Raman map analysis are shown in Figure 4.

The tube presented in Figure 2b is analyzed in terms of the peak position of the LO mode (Figure 4a) and the Raman intensity of the TO mode (Figure 4b). Notice in the map of the LO peak position that, contrary to the case of the cone discussed above, the regions with lowest LO Raman shifts do not match with the regions where the TO mode is observed. So far, the reasons for this observation are unclear. Nevertheless, the TO peak intensity is remarkably high in regions of the tube that show high structural disorder. This reinforces the hypothesis of the link of this mode with collapse of the rolled up structure.

### Heating up: are the tubes stable?

Finally, the effect of temperature was studied on a uniform tube in order to calibrate the temperature-related shifts and to investigate whether or not the structures are stable at temperatures up to 300°C. To the best of our knowledge, the only similar study reported on the temperature stability of rolled up structures was made in Deneke *et al*. [5] and Songmuang *et al*. [9], although in those reports, the sample temperature and peak positions were not determined independently as is the case in the present work.

**Figure 5:**
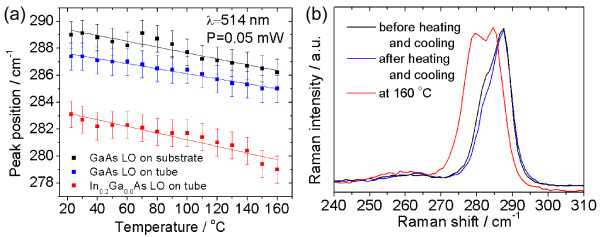
**Thermal investigation under 632.8-nm excitation.** (**a**) Temperature dependence of the LO phonon mode of the GaAs on the tube and on the substrate, respectively. Error bars represent the spectral resolution of ±1 cm^−1^. Panel (**b**) shows the reversibility in the Raman spectra on the tube after heating to 300°C and cooling down again.

**Figure 6:**
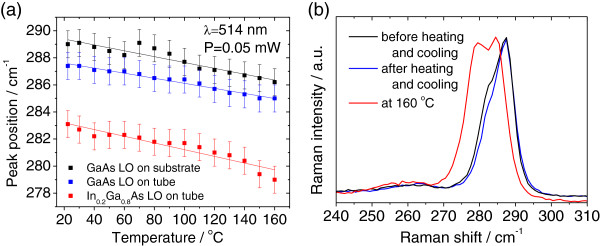
**Temperature dependence of the LO modes and the reversibility of the heating process.** Temperature dependence of the LO modes of the InGaAs/GaAs system (**a**). The error bars of ±1 cm^−1^ represent the spectral resolution. (**b**) The reversibility of the heating process is verified using the 514.5 nm line of an Ar^+^ laser with a power measured at the sample of 50 μW.

Using red excitation (*λ* = 632.8 nm) we observe a linear shift of the LO mode towards lower wavenumbers with increasing temperature as shown in Figure 5a. No irreversible damage (which is expected for 450°C) [10] was detected as shown by the Raman spectra before and after heating (Figure 5b). Only thermal expansion takes place when heating the tubes. Additional verification of the temperature stability was performed using the 514.5 nm laser line of an Ar^+^ laser and tracking the positions of the LO Raman modes of the GaAs substrate and the InGaAs/GasAs bilayer tube as shown in Figures 6a,b.

These observations show that defect-free tubes remain stable at relatively high temperatures (which exceed the temperature range for standard electronic applications of maximum 85°C for the industrial grade) due to the good heat dissipation of the membrane-like tubes.

## Conclusions

Raman spectroscopy with mapping capabilities becomes an interesting tool for the assessment of heterogeneities and fractures in faulty InGaAs/GaAs semiconductor rolled up tubes. The appearance of the TO mode of the GaAs tube is suggested as an indication of defects in these structures due to a further relaxation of the selection rules in regions with high defect concentration, although this requires statement/hypothesis further analysis. Raman spectroscopy could then be used as a failure diagnostic tool in structural characterization. Thermal stability of the structures was verified for temperatures up to 300°C. A linear red-shift in the Raman spectra of the structures has been observed which can be attributed to thermal expansion without involving any damage or degradation of the tubes.

## Competing interests

The authors declare that they have no competing interests.

## Authors' contributions

RDR designed the study, wrote the manuscript, coordinated between all the participants, and took part in the Raman imaging experiments and the data analysis. ES performed all the Raman spectroscopy and imaging experiments, carried out the data analysis, and read and improved the manuscript. DJT grew and processed all the GaAs/InGasAs samples, read the manuscript, and coordinated between participants. DL participated in the manuscript preparation, made corrections to the manuscript, and helped set up the study. ODG participated in the coordination and design of the experiments and read and corrected the manuscript. FS participated in the Raman experiments and data analysis. AM contributed with key discussions and insights on the Raman spectroscopy analysis. OGS, MH, and DRTZ participated in the conception of the project, coordinated among all the participants, and read and improved the manuscript. In addition, DRTZ made significant contributions to the data analysis. All authors read and approved the final manuscript.
